# ‘Measuring’ Physical Literacy and Related Constructs: A Systematic Review of Empirical Findings

**DOI:** 10.1007/s40279-017-0817-9

**Published:** 2017-11-15

**Authors:** Lowri C. Edwards, Anna S. Bryant, Richard J. Keegan, Kevin Morgan, Stephen-Mark Cooper, Anwen M. Jones

**Affiliations:** 1 Cardiff School of Sport and Health Sciences, Cardiff Metropolitan University, Cyncoed Road, Cardiff, CF23 6XD, UK; 2 Faculty of Health, Research Institute for Sport and Exercise, University of Canberra, Canberra, ACT Australia

## Abstract

**Background:**

The concept of physical literacy has received increased research and international attention recently. Where intervention programs and empirical research are gaining momentum, their operationalizations differ significantly.

**Objective:**

The objective of this study was to inform practice in the measure/assessment of physical literacy via a systematic review of research that has assessed physical literacy (up to 14 June, 2017).

**Methods:**

Five databases were searched using the Preferred Reporting Items for Systematic Reviews and Meta-Analyses for Protocols guidelines, with 32 published articles meeting the inclusion criteria. English-language, peer-reviewed published papers containing empirical studies of physical literacy were analyzed using inductive thematic analysis.

**Results:**

Qualitative methods included: (1) interviews; (2) open-ended questionnaires; (3) reflective diaries; (4) focus groups; (5) participant observations; and (6) visual methods. Quantitative methods included: (1) monitoring devices (e.g., accelerometers); (2) observations (e.g., of physical activity or motor proficiency); (3) psychometrics (e.g., enjoyment, self-perceptions); (4) performance measures (e.g., exergaming, objective times/distances); (5) anthropometric measurements; and (6) one compound measure. Of the measures that made an explicit distinction: 22 (61%) examined the physical domain, eight (22%) the affective domain; five (14%) the cognitive domain; and one (3%) combined three domains (physical, affective, and cognitive) of physical literacy. Researchers tended to declare their philosophical standpoint significantly more in qualitative research compared with quantitative research.

**Conclusions:**

Current research adopts diverse often incompatible methodologies in measuring/assessing physical literacy. Our analysis revealed that by adopting simplistic and linear methods, physical literacy cannot be measured/assessed in a traditional/conventional sense. Therefore, we recommend that researchers are more creative in developing integrated philosophically aligned approaches to measuring/assessing physical literacy. Future research should consider the most recent developments in the field of physical literacy for policy formation.

**Electronic supplementary material:**

The online version of this article (10.1007/s40279-017-0817-9) contains supplementary material, which is available to authorized users.

## Key Points

**Table:** 

This article is the first to provide a systematic review of the measure/assessment attempts of the concept of physical literacy and its related constructs (i.e., physical activity and health outcomes) and is the first to suggest that by adopting simplistic and linear methods, physical literacy cannot be measured/assessed in the traditional/conventional sense.
Recommendations for future research include a need for more empirical research on the concept of physical literacy; essentially, there is a need for more research that is open about the definition and philosophical approach used and theories tested.
Future research should measure/assess beyond the constructs of physical proficiencies, and aim to measure/assess physical literacy from a more holistic perspective.

## Introduction

### Background to the Concept of Physical Literacy

In recent years, the concept of physical literacy has gained increasing international political attention and has been integrated into several educational and sport policies [1, 2]. It is proposed that physical literacy influences important health outcomes, such as cardiovascular fitness, strength, motor skills, and obesity status [3], and it is associated with a wide array of behavioral, psychological, social, and physical variables [4]. Consequently, some scholars and educational administrations have proposed that physical literacy is as important to a child’s development as literacy and numeracy [5–7]. While many policy makers and stakeholders currently advocate physical literacy programs and interventions, the definitions of physical literacy adopted by these schemes differ [1, 4], thus causing disparities of how to best operationalize and measure/assess the concept.

A recent systematic review outlined the challenges of many definitions of physical literacy and related constructs currently under debate such as different misinterpretations and lack of consistency with operationalization [4]. Some of these definitions focused solely on the physical and motor competence aspects of physical literacy, including: running speed [8]; motor development [9]; fundamental movement skills (FMS) [10]; and the use of ‘exergaming’ technology as a tool to develop physical competence [11, 12]. Other countries define physical literacy as applying FMS with confidence (Northern Ireland) [5] in a range of multiple environments to benefit the development of the whole person (Physical and Health Education Canada) [13]. A number of related constructs to physical literacy have been previously identified such as physical activity [4]. Importantly, the related constructs describe concepts that were related to, but not synonymous with, physical literacy.

In an effort to summarize and synthesize this literature, Edwards et al. [4] conducted a systematic review of definitions and associations of physical literacy. They found that the majority of papers (70%) adopted a ‘Whiteheadian’ definition of physical literacy and that adopted by the International Physical Literacy Association namely as: “the motivation, confidence, physical competence, knowledge and understanding to value and take responsibility for engagement in physical activities for life” [14]. Specifically, Whitehead’s [14] concept of physical literacy is based on the premise of a holistic individualized journey, with three identified philosophical underpinnings of phenomenology, existentialism, and monism—this differs from many of the competing definitions outlined above, which often do not detail their philosophical underpinnings (see [4, 5, 13, 15]). Recent developments in the field, specifically the work of Dudley et al. [16], acknowledged that while philosophical approaches may differ between public health, sport, and educational policies, there is cohesion within policy about the purpose of physical literacy. Overall, there are inconsistencies in the interpretation and operationalization of physical literacy that have led to a lack of clarity in intervention design [4]. Indeed, these insights emphasize the need for a critical discussion of philosophical paradigms to ensure the conceptualization, measurement, and interventions deployed in different policies are carefully aligned with a specific philosophical approach.

Debates acknowledging these philosophical standpoints have questioned whether physical literacy can be measured/assessed in any conventional sense, or at least what might constitute an appropriate method of collecting empirical data for the study of physical literacy [17], which also aligns a definition and the proposed philosophy [4]. There is also the important point that the three above philosophical standpoints are not intended to be combined, but rather three stand-alone self-contained perspectives on ontology (what is the nature of that reality?) and epistemology (how can we come to know and understand this reality) [18, 19].

In this context, it is important to acknowledge what is meant by measurement/assessment. According to Huitt et al. [20], measurement is the process of quantifying objects/events, and assessment is the process of gathering measurement data to better understand an issue. In qualitative research “measurement is the process observing and recording the observations that are collected as part of a research effort” [21]. For the purpose of this article, the term measuring/assessing was taken to include charting, monitoring, evaluating, characterizing, and/or observing physical literacy, within empirical research studies. Empirical research is one method of gaining a greater understanding of the concept of physical literacy and examining it helps to identify how a concept can be operationalized: i.e., translated from an abstract theoretical concept into a tractable measurable entity. Empirical research is the accumulation of evidence for or against any particular theory, and involves planned experimental or non-experimental designs [22], wherein ‘non-experimental’ can also include qualitative designs. In the present review, empirical data included formal experimentation and non-experimental designs, which included interviews, open-ended questionnaires, reflective diaries, focus groups, participant observations, and visual methods to explore the concept of physical literacy. Experimental empirical studies included a treatment, or intervention, with hypotheses, whereas non-experimental empirical studies include exploratory and observational research such as case studies, surveys, field research, and correlation research [23].

### Grasping the Nettle: Philosophical Assumptions

As noted by Dennett [24]: “There is no such thing as philosophy-free science; there is only science whose philosophical baggage is taken on board without examination”. As such, assumptions about the philosophy of science permeate all science, but are particularly pronounced in the study of physical literacy, as it is proposed from the outset as a concept steeped in philosophical language such as monism, existentialism, and phenomenology [15, 25]. Some philosophers and methodologists insist that it is vital to both declare one’s position prior to engagement with a question/problem, as well as ensuring alignment between ontology, epistemology, and methodology [18, 19]. In this scenario, answers to the questions posed above come as coherent, ‘aligned’ sets, such that decisions regarding ontology determine the most suitable epistemology, and those determine the most appropriate methodology. Others have observed that scientific endeavors can move along without any such efforts, indeed terming this ‘normal’ science [26, 27]. Hassmén et al. [28] have recently made the clear case that failures to acknowledge and address philosophical assumptions are at the heart of a number of tensions and crises within sport and exercise research.

Of course, the decision regarding whether this area of research is ‘in crisis’ is entirely subjective, but in proposing the very concept of physical literacy, Whitehead [15, 25] had ostensibly decided that the confluence of research between physical education (PE), physical activity, health, and motor learning was experiencing a crisis, for example, from inconsistent findings, poor or inconsistent implementation, or falling popular interest/understanding. Furthermore, Whitehead was arguing, both implicitly and explicitly, that a significant portion of this ‘crisis’ was being generated by either inappropriate or missing philosophical assumptions, for example, the seemingly straightforward mechanical assumption that more physical activity in childhood (in both volume and intensity) leads to improved motor skills, which automatically leads to lifelong physical activity, and improved health outcomes. Such an approach would stem from an ‘assumption-set’ termed *positivism*, which asserts that observations made by scientists can and should be completely unbiased and neutral, and that—if sufficient unbiased observations are made—then the underlying mechanisms and explanations will ‘emerge’ and become obvious, leading to theoretical understanding. That understanding can be used to generate specific refined hypotheses, which are then tested in further observations. Implicitly or explicitly, this is the core assumption underlying many scientific studies, even though many of its core assumptions have been disproved [29–31]. It is acknowledged that there are very many different versions of positivism, and indeed post-positivism, but at the broad level the core assumptions remain very similar.

Nevertheless, it is important to ask whether these assumptions are applicable to the concept of physical literacy. Like positivism, several ‘sets’ of assumptions have been proposed arguing that the reality of physical literacy is not the same everywhere, for everyone, and thus cannot be measured in an unbiased, neutral, or consistent way. Broadly classified under the banner of ‘interpretivism’, these approaches rule out both the prospect of objective measurement, and the ‘reduction’ of a complex phenomenon to its component parts for ease of measurement [32]. Fundamentally, this argument is that the focus of physical literacy should be the personal experience: a highly subjective integration of many different experiences spanning physical, emotional, mental, and social phenomena, i.e., the only place all those influences truly ‘integrate’ into a single experience is the individual’s consciousness [15, 25]. In this interpretation, it is unlikely that objective measurement would work, and all we could legitimately attempt would be to track, characterize, and seek to understand each individual’s experience. Notably, all the authors and researchers within these paradigms are emphatic that such an approach is extremely appropriate legitimate science: more legitimate in fact than applying positivist assumptions to such phenomena (see also Gergen’s constructionist work in PE [33–35]). As a final point, other assumption sets exist, including: critical rationalism [30, 31]; critical realism [36, 37]; pan-critical rationalism [38, 39]; and more, but these have not yet been applied in the study of physical literacy.

As a broad summary, two approaches have emerged in relation to how one understands the concept of physical literacy [17]. These approaches are characterized as *idealist* and *pragmatic* perspectives, and have previously been referred to as ‘academic’ and ‘practical’ approaches [40]. An idealist perspective argues that physical literacy is a holistic concept, and therefore the three commonly cited domains of physical literacy (physical, affective, and cognitive) cannot be separated [2]. As such, measuring those domains of physical literacy separately would contradict the holistic philosophical underpinnings of the concept. Consequently, idealists are more likely to explore the concept of physical literacy through qualitative research approaches, such as in-depth interviews, reflections, and observations.

Other scholars have adopted a more pragmatic perspective when seeking to measure the concept of physical literacy [41, 42]. A practical perspective seeks to generate measures that are compatible with evidence-based practice, and contends that research is appraised on its practical implications [43–45]. Pragmatists argue that evidence and practical approaches to the concept of physical literacy are required to change current practices [46]. As a result, pragmatists may choose any methodologies that are compatible with these aims, and are therefore open to using a range of research methods including both qualitative and quantitative [46]. This could be seen as combining the strengths of various methods, yet without critical oversight, it could also be combining the limitations of different approaches. To further complicate this debate, it appears that some researchers adopt a ‘holistic’ definition, yet appreciate the need for an operational (practical) method of measuring physical literacy [4]. Compounding the tensions caused by these conflicting perspectives, there has been an increasing demand for measures/assessments of physical literacy over at least 7 years [47].

A range of initiatives and programs have emerged from the pragmatic approach towards operationalizing physical literacy [1]. Kiwi Sport is an initiative adopted in New Zealand, whereby an emphasis is placed upon non-standardized games, which are used to assess fundamental motor skills [48]. Alternatively, the Scottish ‘Basic Moves’ program evaluates fundamental motor skills through a Test of Gross Motor Development [48]. A criticism of these approaches is that they mainly focus on physical and motor capability, over and above other psychological components of physical literacy. Work in Wales has attempted to measure/assess the physical competence element of physical literacy through a validated ‘Dragon Challenge’ obstacle course [49]. In Canada, an attempt has been made to devise and validate an alternative assessment to capture all elements of physical literacy via the Canadian Assessment for Physical Literacy [41, 42]. This approach attempted to identify the current and most favored measurement approaches for each recognized component of physical literacy, competence, confidence, motivation, and knowledge, but has been criticized for treating them as quite separate, and still providing a disproportional focus on physical and motor competence. Some national ‘Report Cards on Physical Activity in Children and Youth’ have acknowledged physical literacy as an indicator, however, some countries have expressed that data on physical literacy are ‘insufficient’ to provide an overall grade [50, 51].

Further, the importance of physical literacy has been acknowledged by the United Nations Educational, Scientific and Cultural Organisation to generate healthy, able, and active citizens as an outcome of high-quality PE [52]. While it is encouraging that the physical literacy agenda is advancing on a practical level, the degree to which these measurement/assessment attempts capture the multifaceted and relatively unique characteristics of physical literacy remains questionable [53]. The current physical literacy initiatives have centered on children and youth populations, with very little focus on pre-adolescent and adult populations. Further, alignment between definition, philosophy, and measures of physical literacy are yet to be explored [4].

Overall, the tension appears to be between the desire to develop consistent, reliable, and valid measures of physical literacy, vs. the viewpoint that physical literacy is inherently complex and dynamic and thus not readily measured using such instruments. We do not currently know what measures/assessments are most appropriate for different age groups and environments. To help resolve this tension at the heart of physical literacy research, a systematic review of current empirical research—including methods of measuring/assessing the concept of physical literacy—was conducted to facilitate new insight and clarify key considerations. Previous narrative reviews on physical literacy outlined the importance of assessing participants’ knowledge within the concept of physical literacy [54], and emphasized the current lack of robust empirical tools to assess physical literacy [48, 55]. It is important to note, however, that these reviews have not focused solely on measurement attempts, nor were they conducted using a transparent systematic process. Recent attention has emphasized the benefits of systematic reviews, which provide rigorous and transparent methods as a means of minimizing bias and offering a complete coherent overview of contemporary knowledge on a topic [56, 57]. While rigorous and transparent, the analytic steps and presentation of findings in systematic reviews can vary, to address “research questions in different ways with each method” [58].

Two systematic reviews concerning physical literacy and its related constructs have been conducted; specifically, one that investigated the effectiveness of school-based physical activity interventions on students’ health-related fitness knowledge [59] and another that examined the definitions, underlying philosophy, and hypothesized associations/correlates of physical literacy [4]. The findings of the latter suggest a need to operationalize physical literacy as clearly as possible to generate contextualized interpretable (i.e., meaningful) findings. Identifying the similarities and differences in approaches to conceptualizing (and subsequently measuring/assessing) physical literacy will facilitate a degree of pluralism wherein different ideas can compete and be evaluated over time [4]. Accordingly, this development of different well-articulated frameworks for studying physical literacy, if achieved, will allow scholars to decipher which interpretation of physical literacy is being tested, supported, or refuted [4]. In turn, practitioners and policy makers can evaluate the impact of their physical literacy interventions through physical literacy measures/assessments. To date, no systematic review has focused on empirical studies of physical literacy and the attempts made therein to measure/assess physical literacy.

### Purpose and Objectives

The purpose of this systematic review was to collate and analyze empirical studies conducted on physical literacy and its related constructs, and to synthesize, and reflect on, current (up to 14 June, 2017) empirical measurement practice regarding physical literacy. Consequently, the aim will be met through the following two objectives:

1. To systematically review the empirical research and measurement/assessment attempts in relation to the concept of physical literacy and its related constructs (e.g., physical activity/health outcomes); and
2. To critically characterize, evaluate, and compare existing measures/assessments of physical literacy and its related constructs in relation to age group, environment, and philosophy.

## Methods

The methodology of this article was adapted from that of Edwards et al.’s [4] systematic review on the definitions, foundations, and associations of physical literacy, which used the Preferred Reporting Items for Systematic Reviews and Meta-Analyses for Protocols (PRISMA-P) [60], and deployed thematic analysis for evaluating and organizing the findings.

### Information Sources and Search Strategy

An electronic search strategy was deployed, using the following databases: (1) SPORTDiscus; (2) MEDLINE (via PubMed); (3) Scopus; (4) ScienceDirect; and (5) Education Research Complete, last searched on 14 June, 2017. The above databases report on areas including education, sport, and health, which are relevant to the concept of physical literacy and therefore increased the likelihood that all relevant studies were located [61, 62]. A Boolean logic combinations search strategy was adopted within the electronic databases, including “physical literacy” with measurement, assessment, charting, monitoring, evaluation, test, analysis, case study, practical, applied, intervention, trial, predictor, correlation, association, and relationship. Inverted commas were applied to the term “physical literacy” to ensure searches would find papers in relation to physical literacy as opposed to searches related to ‘physical’ and ‘literacy’. English language, peer-reviewed, and journal filter boxes were marked on all searches to ensure only these papers would appear in the results [see Appendix S1 of the Electronic Supplementary Material (ESM)]. It was not possible to apply these filters or to use Boolean phrases in Google Scholar; therefore, the latter was not used in this study. Additional records were selected through identifying sources from the reference lists of the records identified through database searching [60].

### Eligibility Criteria and Study Records

**Fig. 1:**
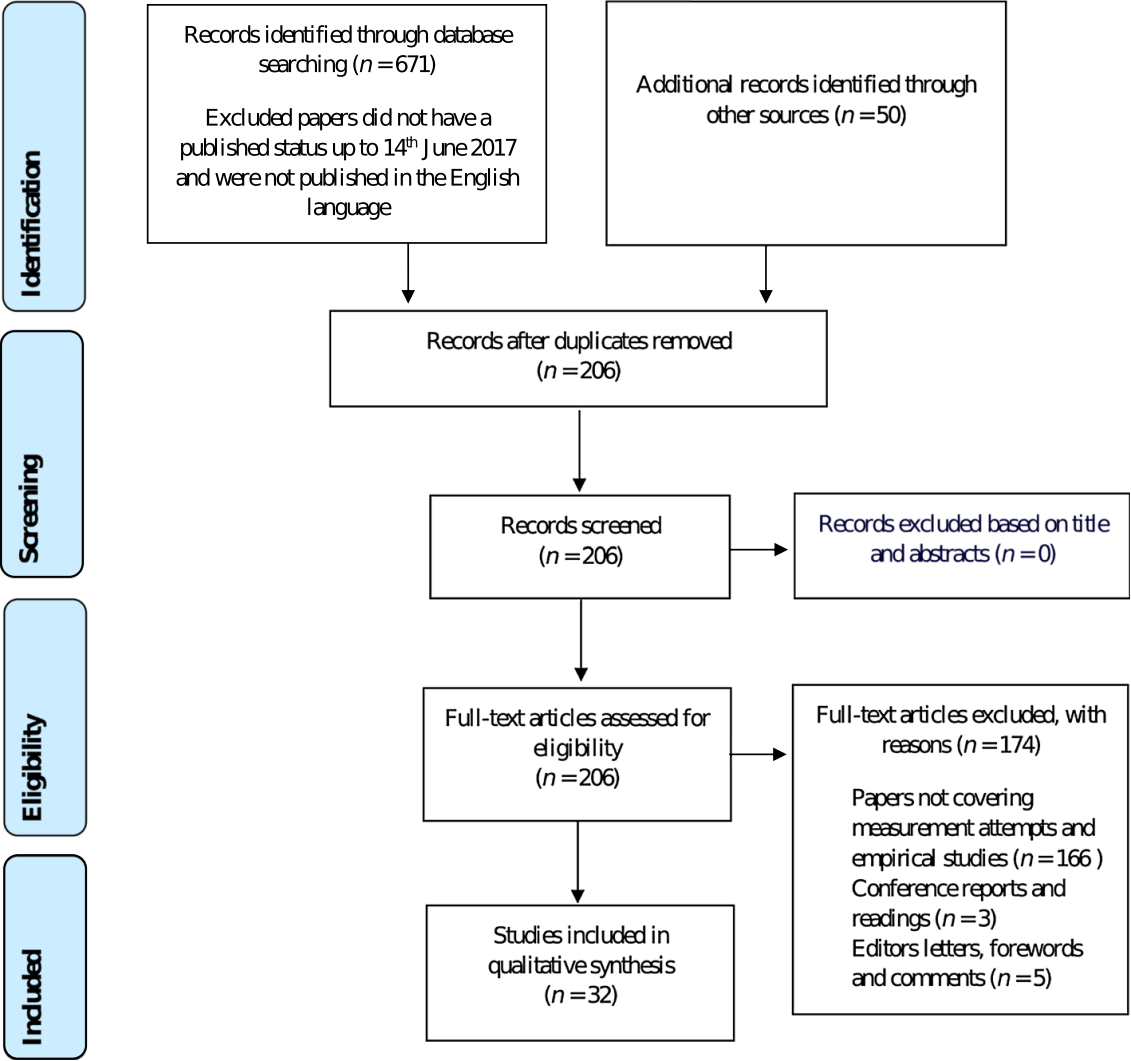
Preferred Reporting Items for Systematic Reviews and Meta-Analyses flow diagram [60]

The inclusion criteria in this systematic review were as follows: (1) papers with a peer-reviewed published status; and (2) publications in the English language up until the date last searched: 14 June, 2017. To address the aims and objectives of the study, the following exclusion criteria were adopted: (1) papers not attempting to measure/assess attempts and/or empirical studies; (2) conference reports and readings; and, (3) editors’ letters, forewords, and comments. The authors used the PRISMA-P evidence-based checklist during the planning, conduct analysis, and reporting of this process [60]. The PRISMA-P flow diagram for this study can be found in Fig. 1 and the PRISMA-P checklist can be found in Appendix S2 of the ESM [60].

A total of 671 papers were identified through database searches and an additional 50 records were retrieved from the reference lists in these 671 papers. In line with the PRISMA-P procedures, a total of 515 duplicated papers were removed during the search process, leaving 206 papers for the screening process. Non-duplicated papers were read thoroughly by two analysts and evaluated against the inclusion/exclusion criteria: in each case, mutual agreement was required between analysts [63]. To ensure consistency between analysts, a calibration exercise was conducted before commencing the data collection. During the selection process, the analysts uploaded their literature search results to a shared electronic file in an attempt to reduce publication and selection bias. Any discrepancies between the two analysts were resolved by consensus and/or discussion with a third investigator. Records were kept of this process with an 89% agreement prior to discussion and a 100% agreement post-discussion. To assess the possible risk of bias in individual studies, the analysts adhered to the Cochrane Collaboration tool for assessing the risk of bias, which included identifying a low and high risk of bias for the following criteria: sequence generation, allocation concealment, blinding, incomplete outcome data, and selective outcome reporting [64]. During the data analyzing process, the following roles were adopted: (1) the analyst (who was also the first author; LE); (2) one co-analyst (AB); (3) one consensus validator (KM); (4) two internal critical friends (S-MC and AJ); and, (5) one external critical friend (RK). After this thorough process, and consistent with the exclusion and inclusion criteria, a total of 32 papers were included in the review (see Fig. 1).

### Data Items and Data Synthesis

Initially, inductive thematic analysis was employed to extract, label, and evaluate data from each paper. Characteristics including the author(s), definition of physical literacy used, philosophy adopted, outcome assessed, strengths and limitations of measures/assessment in relation to physical literacy and related constructs, age group, and environment were extracted from the 32 papers in the analysis (see Tables 2, 3, 4). The purpose of this process was to summarize the key features of each paper prior to conducting the thematic analysis. The process of thematic coding focused on unfolding both implicit and explicit ideas within the data [65]. Subsequently, qualitative synthesis using thematic analysis was performed on data from the 32 included papers. Thematic analysis was employed to distinguish common categories through analytical examination and recording themes within the 32 papers included in the analysis with the main purpose of data retrieval [66, 67].

To allow replication and transparency of data synthesis, a two-step process was performed. First, basic coding techniques to identify the general themes were completed, followed by interpretative coding that emphasized specific themes in the data [67]. This process comprised organizing themes into: (1) two higher order themes; (2) ten sub-themes; and (3) 52 measures/assessments (see Table 1) [66]. Table 1 displays the hierarchical structure that allows clear identification of the development from a core category to a sub-theme on to a higher order theme as well as identifying the frequency of each core category (i.e., how many papers referred to this item). The Grading of Recommendation, Assessment, Development and Evaluation approach was applied in the present study to provide a transparent guide on rating the quality of research [68]. This included incorporating appropriate items for qualitative synthesis under the following five headings: risk of bias, inconsistency, indirectness, imprecision, and publication bias [69].

**Table 1:** Thematic analysis of the measures/assessments of physical literacy and its related constructs

Higher order themes	Sub-themes	Measures/assessments
Qualitative	Interviews (8)^a^	Different environments (schools, universities, retirement homes) (5)
		Determine the effectiveness of interventions (2)
		Students’ and teachers’ perceptions of inclusive PE (1)
	Open-ended questionnaires (4)	Pupil attitude, opinion, and knowledge of PE (3)
		Willingness of PE teachers to apply physical literacy (1)
	Reflective diary (4)	Teacher reflections on the effectiveness of PE (1)
		Pupil reflections of food consumption (1)
		Pupil reflections to set individual physical activity targets (1)
		Student written responses to daily journal prompts (1)
	Focus groups (4)	Role of play in physical literacy from a child’s perspective (1)
		Students’ perceptions of ability, disability, and inclusion in PE (1)
		Retired people’s understanding of physical literacy (1)
		PE specialist primary and secondary teachers (1)
	Participant observation (4)	Children’s interactions with the outdoor environment (2)
		Social interactions between retired people (1)
		Phenomenological observations of children (1)
	Visual methods (8)	Photo elicitation (2)
		Video recordings (5)
		Portfolio (1)
Quantitative	Physical domain (31)	Accelerometer (2)
		Exergaming (2)
		International Physical Activity Questionnaire (2)
		Pedometer (4)
		Postural tests (2)
		20-m multi-stage fitness test (1)
		Anthropometric measures (2)
		Bruininks–Oseretsky Test of Motor Proficiency (1)
		FMS-Polygon (1)
		Henderson and Sugden’s Movement Assessment Battery for Children (1)
		Agility test (2)
		Non-validated battery of six motor tests (1)
		Nutrition and Physical Activity Self-Assessment of Child Care (1)
		Perceptions of Physical Activity Importance and their Children’s Ability Questionnaire (1)
		Performance diary (1)
		Physical Activity Questionnaire for Older Children (1)
		System for Observing Fitness Instruction Time (1)
		Straight sprint test (1)
		Taco Bell Challenge (1)
		Test of gross motor development (1)
		The Canadian Agility and Movement Skills Assessment (1)
		Vertical jump (1)
	Affective domain (8)	Brustad’s Children’s Attraction to Physical Activity Scale (1)
		Children’s Physical Activity Self-Efficacy Scale (1)
		Children’s Self-Perception of Adequacy in and Predilection for Physical Activity Scale (1)
		Global Physical Self-Worth subscale of the Child and Youth Physical Self-Perception Profile (1)
		Harter’s Self-Perception Profile for Children (1)
		Intrinsic Motivation Inventory (1)
		Non-validated affective questionnaire (1)
		Physical Ability subscale of the Self-Description Questionnaire (1)
	Cognitive domain (5)	Creative thinking test (1)
		Mock exam paper (1)
		Non-validated cognitive questionnaire (1)
		Optional creative writing assignments (1)
		Understanding physical literacy questionnaire (1)
	Physical, cognitive, and affective (2)	The Canadian Assessment for Physical Literacy (2)

*FMS* fundamental movement skills, *PE* physical education

^a^Numbers in parenthesis represent the number of papers that referred to the core categories apparent out of a possible 32 papers

## Results

### Summary of Studies

The papers that were identified, screened, and considered for eligibility are summarized in Fig. 1 [60]. Table 1 provides an overview of the core categories, sub-themes, and higher order themes that were evidenced from the analysis. Table 2 provides critical analyses of the qualitative measures/assessments used in the 32 studies in relation to physical literacy and its related constructs. Table 3 characterizes, evaluates, and compares existing qualitative measures/assessments of physical literacy and its related constructs in relation to age group, environment, and philosophy. Finally, Table 4 provides critical analyses of the quantitative measures/assessments.

**Table 2:** Critical analysis of the qualitative measures/assessments of physical literacy and its related constructs

Measures/assessments (number of papers)	Definition(s) of physical literacy used	Philosophy adopted	Outcome assessed	Strengths of measure/assessment in relation to physical literacy and related constructs	Limitations of measure/assessment in relation to physical literacy and related constructs
Interviews (9)	Whiteheadian definition [70, 73, 74, 77, 78, 82, 83] Developing literacy skills [84] PHE Canada definition [80, 84]	Holistic philosophy [70, 74, 78, 80, 82, 83] No philosophy [73, 77, 84]	To gauge the effectiveness of physical literacy interventions To explore the movement experiences of children and adults Opinions about the efficiency of physical literacy	Allows for exploration and in-depth responses around the cognitive (knowledge and understanding) domain of physical literacy Captures thoughts and feelings of a physical literacy intervention Can be used to capture multiple individuals’ understanding of physical literacy and interventions of physical literacy, i.e., pupils and teachers Able to discuss the affective domain of motivation and confidence	Cannot capture the physical domain of physical literacy, although perceived physical competence could be captured Unable to capture social interactions/interactions with the physical environment
Open-ended questionnaires (4)	Whiteheadian definition [71, 73, 75, 79]	No philosophy [71, 73, 75, 79]	To explore children’s perceived competence and attitudes towards PE To investigate PE teachers’ and pupils’ understanding of physical literacy To determine expert opinions to evaluate physical domain assessments	Captures thoughts and feelings of a physical literacy intervention Allows for exploration and detailed, in-depth responses around the cognitive (knowledge and understanding) domain of physical literacy Able to discuss the affective domain of motivation and confidence Can be used to enhance responses in interviews and focus groups	Knowledge and understanding/cognitive and writing ability is dependent on the age and ability of the participants Unable to capture the physical domain of physical literacy, although perceived physical competence could be captured Cannot capture social interactions/interactions with the physical environment
Reflective diary (4)	Whiteheadian definition [42, 73, 74, 80]	Holistic philosophy [74, 80] No philosophy [42, 73]	To attempt to monitor effective teaching strategies or experiences Recording of performances and self-rating of effort and improvement Explore movement experiences of children and adults	Captures individual thoughts and feelings of a physical literacy intervention Allows for exploration and responses in a detailed, in-depth manner around the cognitive (knowledge and understanding) domain of physical literacy Able to discuss the affective domain of motivation and confidence Can be used to enhance responses in interviews and focus groups	Knowledge and understanding/cognitive and writing ability is dependent on the age and ability of the participants Unable to capture the physical domain of physical literacy, although perceived physical competence, perceived effort/exertion and improvement could be captured Unable to capture social interactions/interactions with the physical environment
Focus groups (4)	Whiteheadian definition [72, 77, 78, 81]	Holistic philosophy [72, 78, 81] No philosophy [77]	To explore the movement experiences from a child’s perspective To determine expert opinions to formulate a quantitative survey To gauge the effectiveness of physical literacy interventions	Captures social interactions with other individuals Allows for exploration detailed, in-depth responses around the cognitive (knowledge and understanding) domain of physical literacy Able to discuss the affective domain of motivation and confidence	Cannot capture interactions with the physical environment Unable to capture the physical domain of physical literacy Responses can be influenced by other members of the focus group and goes somewhat against the concept of an individual journey
Participant observation (5)	Whiteheadian definition [76, 77, 80, 82] No definition [88]	Holistic philosophy [76, 80, 82] No philosophy [71, 88]	To ascertain the amount of time participants were active in their learning/engaging with physical activity To explore phenomenological observations from the child’s perspective	Captures social interactions/interactions with the physical environment Captures the physical domain of physical literacy Able to observe one’s level of engagement in physical activities	Cannot capture in-depth responses around the cognitive (knowledge and understanding) domain of physical literacy Unable to capture an individual’s understanding of physical literacy/interventions of physical literacy Cannot capture thoughts and feelings of a physical literacy intervention Unable to observe the internalized affective domain of motivation and confidence Some participants may try harder if they know they are being observed
Photo elicitation (2)	Whiteheadian definition [72] Developing physical skills [85]	Holistic philosophy [72] No philosophy [85]	Understand the role of active play in promoting physical literacy from a child’s perspective Food intake using digital photography-weighted plate waste method	Can be used to enhance responses in interviews and focus groups Captures aspects of the participants’ interactions with the physical environment Captures the participants’ knowledge and understanding of what physical literacy means to them in a pictorial form	Unable to capture an individual’s understanding of physical literacy/interventions of physical literacy Cannot capture thoughts and feelings of a physical literacy intervention Cannot view the internalized affective domain of motivation and confidence Unable to capture interactions with the physical environment Unable to represent the whole environment, i.e., behind the camera
Video recordings (4)	Whiteheadian definition [78, 82, 83] Developing literacy skills [84]	Holistic philosophy [78, 82, 83] No philosophy [84]	Captures the physical context and used to capture different observations when multiple activities occur To record interviews	Captures social interactions/interactions with the physical environment Captures the physical domain of physical literacy	Cannot capture in-depth responses around the cognitive (knowledge and understanding) domain of physical literacy Unable to capture an individual’s understanding of physical literacy/interventions of physical literacy Cannot observe the internalized affective domain of motivation and confidence Participants may try harder if they know they are being video recorded
Portfolio (1)	Developing literacy skills [84]	No philosophy [84]	A collection of written accounts of different physical activities completed by students	Allows for detailed in-depth written responses around the cognitive (knowledge and understanding) domain of physical literacy Captures progress over a sustained period of time, reflecting their individual physical activity journey	Knowledge and understanding/cognitive and academic writing ability is dependent on the age and ability of the participants Unable to capture the internalized affective domain of motivation and confidence Cannot capture an individual’s understanding of physical literacy/interventions of physical literacy Problematical to capture social interactions/interactions with the physical environment

*PHE* physical and health education, *PE* physical education

**Table 3:** Characteristics of the qualitative measures/assessments of physical literacy/related constructs characterized under age group, environment, and philosophy

Qualitative	Papers (*n*)	Age group			Environment			Philosophy	
		Children (U12)	Adolescents	Adults	PE	Community	Other	Holistic^a^	No declared philosophy
Interviews	9	5	2	3	6	1	2	6	3
Open-ended questionnaires	4	2	1	1	3	0	1	0	4
Reflective diary	4	4	0	0	2	1	1	2	2
Focus groups	4	1	0	3	2	0	2	3	1
Participant observation	5	3	1	2	3	1	1	3	2
Photo elicitation	2	2	0	0	0	0	2	1	1
Video recordings	4	1	2	1	3	0	0	3	1
Portfolio	1	0	1	0	1	0	0	0	1

*PE* physical education, *U12* under 12 years of age

^a^Holistic philosophy included authors using one or more of the following keywords: whole person, phenomenology, existentialism, monism, holistic

**Table 4:** Critical analysis of the quantitative measures/assessments of physical literacy and its related constructs

Measures/assessments (no. of papers)	Definition(s) of physical literacy	Age group (children U12, adolescents, adults)	Environment (PE, community, other)	Philosophy alignment	Outcome measures assessed (physical literacy domain/related construct)	Strengths of measure/assessment in relation to physical literacy and related constructs	Limitations of measure/assessment in relation to physical literacy and related constructs
Pedometer (4)	No definition [41, 93] Whiteheadian definition [42] Developing physical skills [85]	Children U12 and adolescents	PE and community	No philosophy [41, 42, 85, 93]	Physical activity (related construct of physical literacy)	Accurate, cost effective, and easy to use	Omit the intensity and type of physical activity undertaken
Accelerometer (2)	Developing physical skills [85] No definition [93]	Children U12 and adolescents	Community and PE	No philosophy [85, 93]	Physical activity (related construct of physical literacy)	Details of the intensity and type of physical activity undertaken	Expensive equipment and does not account for water-based activities
International Physical Activity Questionnaire (IPAQ) (2)	Developing physical skills [85] No definition [93]	Children U12	Community and PE	No philosophy [85, 93]	Physical activity (related construct of physical literacy)	Validated questionnaire to gauge physical activity levels	Long and short versions of IPAQ, no detail of the appropriateness of both versions
Nutrition and Physical Activity Self-Assessment of Child Care (NCAP SACC) (1)	Developing physical skills [85]	Children U12	Community	No philosophy [85]	Physical activity (related construct of physical literacy)	The NCAP SACC is completed by two research assistants, which allows consistency with reporting the data	The 55-item questionnaire takes a long time to complete, which may reduce the reliability of the data Two research assistants is unrealistic in a school /club setting
Perceptions of Physical Activity Importance and their Children’s Ability Questionnaire (1)	Developing physical skills [85]	Children U12	Community	No philosophy [85]	Physical activity (related construct of physical literacy)	Obtaining parental views on their child’s physical activity ability increases the reliability as the sample of children were aged 3–5 years	Reliant on significant others commenting on children’s physical activity level
Physical Activity Questionnaire for Older Children (1)	No definition [87]	Adolescents	PE	No philosophy [87]	Physical activity (related construct of physical literacy)	Use of recall cues such as play time, PE lessons, after school to prompt children’s recall	Cognitive recall abilities in self-report measures are unreliable
System for Observing Fitness Instruction Time (1)	No definition [88]	Children U12	Community	No philosophy [88]	Physical activity (related construct of physical literacy)	Validated test that is easy to administer and cost effective	Does not capture quality movement, numeric data collected on time spent on moderate-to-active physical activity
Exergaming (2)	Whiteheadian definition [77] PHE Canada definition [12]	Children U12 and adults	PE and community	Reference to holistic philosophy [12] No philosophy [77]	FMS (part of the physical domain of physical literacy)	A viable option for gauging individual physical progress for the future technological generation Reading the environment Alignment with the unique physical literacy journey	Physically competent individuals should interact with a variety of physical environments to develop simple, combined, and complex movement capacities, which could be limited while exergaming
FMS-Polygon (1)	No definition [91]	Children U12	Other	No philosophy [91]	FMS (part of the physical domain of physical literacy)	Allows learners to perform tasks in an applied environment Different aspects of FMS measured/assessed through an obstacle course Cost and time effective and can be effectively administrated in a school or sports club setting	Outcome was measured quantitatively for each task with a time Omits consideration for quality of movement
Henderson and Sugden’s Movement Assessment Battery for Children (1)	Northern Ireland definition [89]	Children U12	Community	No philosophy [89]	Limb positioning, balance and fluency of movement (part of the physical domain of physical literacy)	Ability to evaluate motor development, develop motor training program, and assists in diagnosing motor impairment	Time consuming to administer Does not allow children to display their applied physical competence as skills are completed in isolation
The Canadian Agility and Movement Skills Assessment (1)	Whiteheadian and PHE Canada definition [42]	Children U12	PE	No philosophy [42]	Physical competence (part of the physical domain of physical literacy)	Combines both quality and quantity indictors to measure/assess physical competence Applies Whitehead’s theoretical constructs of physical competence	Lack of detail of the procedures, inability to determine whether the assessment is used in an applied or isolated manner
Postural tests (2)	Whiteheadian definition [92] PHE Canada definition [12]	Children U12 and adults	PE and other	Reference to holistic philosophy [12] No philosophy [92]	Stability/ balance (part of the physical domain of physical literacy)	Use of rigorous and validated methods to collect data	Expensive equipment, which poses problems with accessibility Requires scholarly expertise to analyze data
Test of gross motor development (1)	Developing physical skills [85]	Children U12	Community	No philosophy [85]	Physical literacy (part of the physical domain of physical literacy)	Validated and standardized test designed to assess the gross motor functioning (locomotor and object control skills) of children aged 3–10 years Could be used as part of a larger assessment program	Does not allow children to display their applied physical competence as skills are completed in isolation
Bruininks-Oseretsky Test of Motor Proficiency (1)	Northern Ireland definition [89]	Children U12	Community	No philosophy [89]	Limb positioning, balance and fluency of movement (part of the physical domain of physical literacy)	Ability to evaluate motor development, develop motor training program, and assists in diagnosing motor impairment	Time consuming to administer Does not allow children to display their applied physical competence as skills are completed in isolation
Straight sprint test (1)	Developing physical skills [90]	Children U12	Community	No philosophy [90]	Motor performance (part of the physical domain of physical literacy)	Time effective Could be used as part of a larger assessment program	Measures speed only
Vertical jump test (1)	Developing physical skills [90]	Children U12	Community	No philosophy [90]	Motor performance (part of the physical domain of physical literacy)	Time effective Could be used as part of a larger assessment program	Measures explosive power only
Agility test (2)	Developing physical skills [90]	Children U12	Community	No philosophy [90]	Motor performance (part of the physical domain of physical literacy)	Time effective Could be used as part of a larger assessment program	Measures agility only
20-m multi-stage fitness test (1)	Whiteheadian definition [73]	Children U12	PE	No philosophy [73]	Cardiovascular endurance (related construct of physical literacy—health and fitness)	Validated test that is easy to administer and cost effective	It is often administered in a comparative way as opposed to focusing on personal best, which contradicts the concept of physical literacy
Anthropometric measures (2)	No definition [41] Developing physical skills [90]	Children U12	Community and other	No philosophy [41, 90]	Height, weight, BMI, waist circumference (related construct of physical literacy—health and fitness)	Validated test that is easy to administer and cost effective	May impact on one’s self-esteem
Non-validated affective questionnaire (1)	Whiteheadian definition [71]	Children U12	PE	No philosophy [71]	Perceived competence in PE (part of the affective domain of physical literacy)	Allows teachers to identify useful information regarding pupils’ attitudes and opinions toward PE and physical activity	Cognitive recall abilities in self-report measures can be unreliable Non-validated and therefore unreliable
Brustad’s Children’s Attraction to Physical Activity Scale (1)	Northern Ireland definition [89]	Children U12	Community	No philosophy [89]	Self-perceptions and attitudes towards physical activity and sport (part of the affective domain of physical literacy)	Gauges children’s attitudes toward physical activity, which may assist in planning physical literacy interventions	Lack of an explicit connection with motivation and confidence
Children’s Physical Activity Self-Efficacy Scale (1)	No definition [87]	Adolescents	PE	No philosophy [87]	Self-efficacy (part of the affective domain of physical literacy)	Provides an understanding of a child’s self-efficacy, linking to the confidence element of physical literacy	Self-efficacy is only one part of the affective domain of physical literacy
Children’s Self-Perception of Adequacy in and Predilection for Physical Activity Scale (1)	Whiteheadian and PHE Canada definition [42]	Children U12	PE	No philosophy [42]	Perceived competence (part of the affective domain of physical literacy)	Assesses a child’s desire to participate in physical activities, enjoyment, and perceived physical competence, which is vital to gauge confidence and motivation Assessing children’s self-perceptions may assist with planning physical literacy interventions	Cognitive recall abilities in self-report measures are unreliable
Global Physical Self-Worth subscale of the Child and Youth Physical Self-Perception Profile (1)	No definition [87]	Adolescents	PE	No philosophy [87]	Self-esteem (part of the affective domain of physical literacy)	Assessing children’s self-worth may assist with planning physical literacy and well-being interventions	Lack of an explicit connection with motivation and confidence
Performance diary (1)	Whiteheadian definition [74]	Children U12	PE	Reference to holistic philosophy [74]	Physical competence, self-rated effort and improvement (part of the affective domain of physical literacy)	Child-centered approach to assessing perceived physical competence	Written quality is dependent on children’s academic ability as to the amount of detail captured
Harter’s Self-Perception Profile for Children (1)	Northern Ireland definition [89]	Children U12	Community	No philosophy [89]	Self-perceptions and attitudes towards physical activity and sport (part of the affective domain of physical literacy)	Provides an understanding of a child’s self-esteem, linking to the confidence element of physical literacy	Lack of an explicit connection with motivation and confidence
Intrinsic Motivation Inventory (IMI) (1)	Whiteheadian definition [74]	Children U12	PE	Reference to holistic philosophy [74]	Motivation, enjoyment, perceived competence and effort (part of the affective domain of physical literacy)	Full IMI is validated for adults and is widely accepted to assess individual’s intrinsic motivation Adapted IMI content was appropriate for children	Children’s recall abilities in self-report measures are unreliable The adapted IMI for children is not completely validated
Physical Ability subscale of the Self-Description Questionnaire (1)	No definition [87]	Adolescents	PE	No philosophy [87]	Perceived competence (part of the affective domain of physical literacy)	Physical self-worth affects global self-efficacy, and self-esteem indivisibly connects with motivation	Children’s recall abilities in self-report measures are unreliable
Torrance Test of Creative Thinking (1)	Developing physical skills [90]	Children U12	Community	No philosophy [90]	Creative thinking (part of the cognitive domain of physical literacy)	Validated and reliable instrument Creativity in interacting with the physical environment integral to the concept of physical literacy	Does not evaluate pupils’ general knowledge and understanding of healthy and active lifestyles
Mock exam paper (1)	No definition [86]	Adolescents	PE	No philosophy [86]	A level pupils’ PE knowledge (part of the cognitive domain of physical literacy)	Aimed to improve pupils’ knowledge and understanding of the PE curriculum content	Does not evaluate pupils’ general knowledge and understanding of healthy and active lifestyles
Understanding physical literacy questionnaire (1)	Whiteheadian definition [79]	Adults	Other	Reference to holistic philosophy [79]	Measure Perceived Physical Literacy Instrument for physical education teachers (part of the cognitive domain of physical literacy)	Questionnaire has undergone a rigorous process to validate the questionnaire by a panel of experts in the areas of sport science, physical education, and health education	Not all 18 items on the instrument were applicable to understanding physical literacy
Non-validated cognitive questionnaire (1)	Whiteheadian definition [75]	Children U12, adolescents and adults	PE	No philosophy [75]	PE teachers’ and pupils’ understanding of physical literacy (part of the cognitive domain of physical literacy)	Identifies teachers’ and pupils’ understanding of physical literacy	Cognitive recall abilities in self-report measures are unreliable Non-validated instrument
Optional creative writing assignments (1)	Whiteheadian and PHE Canada definition [80]	Children U12 and adults	Other	Holistic philosophy [80]	Creative assignments of their physical experiences (part of the cognitive domain of physical literacy)	Creative writing assignments allowed researchers to familiarize with students’ perspective, knowledge and understanding	Process of analyzing the creative writing assignments is time consuming Dependent on academic writing ability
Canadian Assessment for Physical Literacy (CAPL) (2)	Whiteheadian definition [42, 79]	Children U12 and adults	PE and other	No philosophy [42, 79]	Physical literacy (physical, affective and cognitive domains of physical literacy and related construct of physical literacy)	The first pragmatic measure that attempts to capture all domains of physical literacy in a measurement/assessment tool Recommendations of the CAPL were made by an international expert panel	The weighting prioritizes the physical domain Normative data as opposed to focusing on personal best

*BMI* body mass index, *FMS* fundamental movement skills, *IMI* Intrinsic Motivation Inventory, *PE* physical education, *PHE* physical and health education, *U12* under 12 years of age

Two higher order themes were distinguished: qualitative approaches and quantitative approaches. For the qualitative higher order theme, 19 core categories were evidenced under the following six subthemes: interviews, open-ended questionnaires, reflective diaries, focus groups, participant observation, and visual methods. For the quantitative higher order theme, 36 core categories were evidenced under the following four sub-themes: (1) physical domain; (2) affective domain; (3) cognitive domain; and (4) physical, affective, and cognitive domains (see Table 1).

As illustrated in Table 2, it was evident that 83% of qualitative papers used a Whiteheadian definition of physical literacy in their measures/assessments [42, 70–83]. The remaining 17% of papers measured/assessed physical literacy by defining physical literacy as either: (1) developing literacy skills in a physical environment [84]; (2) developing physical competency skills [85]; (3) adopting the Physical and Health Education Canada definition [80]; or (4) not declaring a specific definition [86].

Overall papers measuring/assessing the physical domain were distributed reasonably equally across the different environments, namely: four measures/assessments took place in PE lessons [12, 42, 73, 87]; four in the community [76, 88–90]; and five in other environments [41, 85, 91–93] (see Table 4). Four measures/assessments of the affective domain were conducted within PE lessons [42, 71, 74, 87] and one measure/assessment in the community [89]. Two measures/assessments of the cognitive domain were undertaken within PE lessons [74, 86]: two in the community [80, 90] and one in a research-based environment [79].

## Discussion

There is limited empirical research that has attempted to measure/assess physical literacy to date. Papers that included any element of physical literacy and its related constructs, such as physical activity, were therefore included in the analysis. The analysis identified a total of 78 codes, which were organized into 55 core categories and ten sub-themes. These were then organized into two higher themes to address the study’s aims and objectives. The following section will review these two higher themes: qualitative and quantitative measures/assessments.

### Qualitative Measures/Assessments

Many qualitative methods allowed researchers to gain in-depth responses to measure/assess the cognitive and/or affective domains of physical literacy. For example, interviews, open-ended questionnaires, reflective diaries, focus groups, and portfolios could measure/assess individuals’ motivation and confidence towards participating in physical activity, as well as provide opportunities to gauge knowledge and understanding of physical activity and healthy lifestyle behaviors [4]. Interviews, open-ended questionnaires, reflective diaries, focus groups, and portfolios were, however, unable to measure/assess an individual’s physical competence as they are reliant on self-perceptions and/or perceptions of others [70, 73, 74, 77, 78, 80, 82–84]. Indeed, aside from participant observation and video recordings, there were very few qualitative methods that measured/assessed the physical domain of physical literacy [76–78, 80, 82–84, 94]. Using a range of qualitative methodologies and considering all three domains (physical, affective, and cognitive) could address limitations in measuring/assessing physical literacy in a holistic manner [95]. Nonetheless, a crucial point in determining strengths and limitations of qualitative research is the role and quality of the researcher [96]. The interpretive nature of qualitative research could influence the strengths and limitations of methods/results and instigate bias; therefore, caution is required when solely relying on qualitative data.

Another prominent aspect of physical literacy was the social element, i.e., social interactions with peers in the physical environment [15]. Its prominence in physical literacy has prompted some scholars to view ‘social’ as the fourth domain of physical literacy [97]. Some qualitative methods could be used to measure/assess social interactions with peers, namely, focus groups, participant observations, and video recordings. A critique of the current literature is that no measure/assessment to date has attempted to capture the social domain. Nevertheless, some qualitative methods captured interactions with the physical environment, to capture individuals’ responses to “the embodied needs of the perceived environment” (participant observation and video recordings) [15], though most qualitative methods could not capture interactions with the physical environment (interviews, open-ended questionnaires, reflective diaries, focus groups, and photo elicitation) [76–78, 80, 82–84, 94]. Social interactions and interactions with the environment are central to the phenomenological and existential philosophical underpinnings of the concept, as the richer one’s interactions with the environment, the greater one will understand and reach their human potential [4, 98, 99]. As such, using qualitative methods to measure/assess these interactions as part of the wider physical literacy concept attempts to retain the integrity of its holistic nature.

Overall, interviews, focus groups, participant observation, and video recordings were predominantly holistic in their philosophy, whereas open-ended questionnaires and portfolios did not declare a philosophy. More qualitative papers adopted a holistic philosophy, purportedly drawing from phenomenology, monism, and existentialism (*n* = 18) as opposed to not declaring their philosophical assumptions (*n* = 15). In this review, the adoption/declaration of a holistic philosophical standpoint was dependent on the individual studies as opposed to the specific qualitative methodology. To achieve alignment between the definition, philosophy, and outcome measure/assessment, researchers working within physical literacy should be explicit about the definition and philosophy they adopt.

Significantly more qualitative papers measured/assessed physical literacy with children under 12 years of age, compared with adolescents and adults (children aged under 12 years, *n* = 18; adolescents, *n* = 7; adults, *n* = 10). A likely reason for more measures/assessments in children aged under 12 years may be the opportunistic research strategies, as children aged under 12 years are readily accessible in a school environment. The results of the analysis suggest that interviews, reflective diaries, photo elicitation, and participant observation were highly suitable for children aged under 12 years (see Table 3) [42, 70, 72–74, 76, 80, 82, 85]. These qualitative measures/assessments are suitable because they are individualized, which permits a non-comparative experience, thus aligning with the holistic nature of the physical literacy concept and a mastery motivational climate, which emphasizes self-referenced improvement and personal progress as the criteria for success [100].

Nonetheless, children/adolescents’ thoughts and feelings are unpredictable and could change on a daily basis, making it challenging to effectively measure/assess the affective and cognitive domains of physical literacy with qualitative measures/assessments alone. Conversely, open-ended questionnaires, focus groups, and video recordings were not as appropriate for children aged under 12 years [71–73]. Written forms of data such as open-ended questionnaires may elicit in-depth responses from children; however, they are reliant on the academic ability of the child. Therefore, careful consideration of the age/ability of each child is required to determine the appropriateness of open-ended questionnaires. Similarly, the use of video recordings to assess physical competence and interactions with the environment is reliable [101]; however, researchers may face many safeguarding and ethical barriers to video recording children aged under 12 years, as well as a change in normal behavior if children are aware that they are being recorded [82]. This suggests that alternative qualitative measures/assessments of physical competence that are less invasive, such as participant observation, may be more appropriate for children aged under 12 years and adolescents [76, 80, 82].

The analysis revealed that the dominant environment to qualitatively assess physical literacy was during PE lessons (*n* = 12 papers) [42, 71, 73–75, 77, 78, 81–84, 94]. One paper assessed physical literacy in a community sports club setting [80] and five papers assessed physical literacy in ‘other’ environments such as care homes for the elderly, nurseries, and unstructured physical activity/play settings [70, 72, 77, 79, 85]. Given the assessment-based culture in educational settings, it is unsurprising that PE lessons are the dominant environment to empirically measure/assess physical literacy qualitatively. Nonetheless, as the concept of physical literacy extends over the life course, it is problematic that the vast majority of qualitative research is concentrated within a school environment. More qualitative research with young adults, adults, and elderly citizens in different environments is required to better operationalize the concept over the life course.

### Quantitative Measures/Assessments

In contrast to qualitative measures/assessments, the definition of physical literacy adopted by quantitative measures/assessments varied: 29% of measures/assessments used Whitehead’s definition [42, 71, 73–75, 77, 79, 80, 92]; 29% declared no definition [41, 86–88, 91, 93]; 24% defined physical literacy as developing physical skills [85, 89]; 9% adopted the Physical Health Education Canada definition [12, 42, 80]; and a further 9% used Northern Ireland’s definition [89].

Under the physical domain, two quantitative measures/assessments adopted a holistic philosophy [12], whereas the other 19 quantitative measures/assessments under the physical domain declared no philosophy [41, 42, 73, 77, 85, 87–93]. Under the affective domain, significantly fewer quantitative measures/assessments adopted a holistic philosophy (*n* = 1) [74] compared with no declared philosophy (*n* = 7) [42, 71, 87, 90]. Under the cognitive domain, four measures/assessments did not declare a philosophy [75, 79, 86, 90] and one declared a holistic philosophy [80]. Overall, there was an assumption that the philosophical approach in quantitative research was positivism; however, the majority of quantitative measures/assessments did not declare their philosophical standpoint. In turn, most quantitative studies did not align with the holistic philosophy. For example, most measures/assessments in the physical domain evaluated physical competence, FMS, and motor capacities in isolation instead of in applied settings [42, 89, 90, 92] with the exception of the FMS-Polygon [97].

Further, quality of movement was often not measured/assessed in the quantitative studies that captured the physical domain; tests were usually timed, which was problematic for the following two reasons. First, solely timing a test as the main measure omits the opportunity for quality of movement to be captured. Second, these types of times tests have the potential to create a comparative environment if administered in the incorrect manner [42, 73, 85, 90–92]. Consequently, this contradicts the philosophical underpinnings of the concept as there should be a concentration on individualized ability and progress [15]. Separating the individual domains of physical literacy (physical, affective, and cognitive) to measure/assess physical literacy arguably refutes the ‘holistic’ philosophical underpinnings of the concept. Thus, it would be important for those invoking an integrated holistic philosophy to physical literacy to specify how their measurement approaches acknowledge and accommodate this core assumption.

Similar to the findings in the qualitative measures/assessments subsection, children aged under 12 years were the leading age group studied most often under the physical domain with a total of nine quantitative measures/assessments [12, 41, 42, 73, 85, 88–91]. Adolescents [87, 93] and adults [76, 92] had two quantitative measures/assessments, respectively, under the physical domain. Further, more quantitative papers measured/assessed the affective domain of physical literacy in children aged under 12 years (*n* = 4) compared with adolescents (*n* = 1) and adults (*n* = 0) [42, 71, 74, 87, 89]. Children aged under 12 years [75, 80, 90] and adults [75, 79, 80] were the dominant age groups under the cognitive domain with three quantitative measures/assessments, respectively, compared with two measures/assessments with adolescents [75, 86].

Though there are limitations with recall in self-report measures with children, many quantitative measures/assessments across physical, affective, and cognitive domains were judged to be reliable [42, 85, 87–90, 93]. A generic quantitative measure/assessment of physical literacy is not favorable as it would be challenging to integrate all domains and make it relevant for different sports/activities. For example, physical activity for elderly citizens may include gardening, thus a validated tool of their motor proficiency and a questionnaire on their attitude towards physical activity would not provide an accurate representation of their physical literacy journey. Attempting to develop quantitative tools that specify validated ‘ages’ leads to further debate surrounding their appropriateness for physical literacy as the ‘stage not age’ concept departs from normative assessment strategies, which are often employed in the quantitative research measures/assessments [15, 85, 90, 91].

Many quantitative measures/assessments are cost/time effective and easy to administer; therefore, they would be accessible in a variety of different environments (PE/community/other) [41, 42, 73, 85, 93]. Given the recent demand on schools to continually assess learners’ progress, adopting quantitative measures/assessments may help teachers track pupil progress, identify areas for development, and plan interventions tailored to each learner [102]. Using assessment for learning strategies to achieve this would provide a greater focus on formative, as opposed to summative assessment strategies, which is consistent with high-quality PE [103]. Further, quantitative research may evidence the effectiveness of these physical literacy interventions, which in turn may generate funding to conduct further impactful research. Nonetheless, many practitioners would argue that administering and analyzing measures/assessments of physical literacy is unrealistic in educational settings because of teachers’ time constraints, and the current priority of literacy and numeracy [104, 105]. For a viable change to occur on the ground, more accountability for physicality and physical literacy is required in schools so that teachers prioritize physical literacy alongside literacy and numeracy. That said, teachers engaging with the concept of physical literacy should be reminded and assured that measuring/assessing physical literacy quantitatively is not the quintessential component of the concept: i.e., the pedagogical processes that generate motivated, confident, and knowledgeable learners are imperative to engage children in physical activity throughout the life course [106]. If practitioners use measures/assessments without consideration for pedagogy, they are likely to disengage children, thus contradicting the key purpose of the concept [15]. Penney et al. [107], drawing on Bernstein’s [108] conceptualization of curriculum, argue for the inter-relationship between curriculum, assessment, and pedagogy as a fundamental dimension of quality PE.

The results and discussion clearly demonstrate a scarcity of measures/assessments that attempt to capture the entire range of domains within physical literacy, and/or treat it as an integrated construct across these domains. To date, only one measure/assessment (Canadian Assessment for Physical Literacy) has attempted to collectively measure/assess three domains of physical literacy (physical, affective, and cognitive). Three potential reasons for the limited measures/assessments of physical literacy include: (1) researchers are yet to discover measures/assessments that align with their definition and philosophy of physical literacy; (2) researchers are yet to discover the appropriate physical literacy measures/assessments for the age and/or environment; and, (3) the complex and multifaceted physical literacy concept poses challenges to researchers on how it is best operationalized. Adding to this complexity, the results of the present systematic review indicate that researchers are yet to consider the social domain within measures/assessments of physical literacy. As such, our analysis revealed that by adopting simplistic and linear methods, physical literacy cannot be measured/assessed in a traditional/conventional sense. In this context, there is a need for more creative approaches to measure/assess physical literacy through non-conventional methods. Future research should therefore consider the more recent developments by Dudley et al. [16] in the field for physical literacy policy formation in the public health, recreation, sport, and education sectors.

### Limitations

Papers in the English language were solely considered for this systematic review; thus, the papers were primarily derived from the UK and Canada, which may be considered as a limitation. Owing to the limited empirical research on the concept of physical literacy, the 32 papers included in the present study encompassed both physical literacy and its related constructs, such as physical activity. Caution should be exercised when assessing the papers that measure/assess the related constructs of physical literacy. These papers should not be considered as the sole method to measure/assess physical literacy, but used in conjunction with explicit physical literacy assessments. Nevertheless, future empirical research and attempts to measure/assess physical literacy will significantly contribute to the field of physical literacy.

## Conclusions

This paper is the first to provide a systematic review of empirical research efforts to measure or assessment physical literacy, and is the first to systematically reveal that the concept cannot be measured/assessed in a traditional and conventional sense using simplistic and linear methods. This systematic review has identified the strengths and limitations of both qualitative and quantitative approaches to measuring/assessing physical literacy in relation to age group, environment, and philosophy adopted [4]. Quantitative measures/assessments more readily facilitate judgments of reliability, validity, and replicability; however, they are less aligned with physical literacy’s holistic philosophy as defined by Whitehead [14]. Consequently, researchers should declare their definition and philosophy to create an alignment with the measure/assessment selected. Qualitative research aligned more with the holistic philosophical underpinnings of phenomenology, existentialism, and monism than did quantitative research. Qualitative measures/assessments allowed researchers to measure/assess the complex and integrated phenomena, such as interactions with the physical environment, which may lead to more legitimate attempts to quantify physical literacy holistically. Overall, qualitative methods of inquiry have more potential to measure/assess the affective and cognitive domains than the physical domain of physical literacy. As identified by the present analysis, no currently available qualitative technique can adequately measure/assess all physical literacy domains, particularly in a way that reflects the integrated non-linear nature of the concept. Therefore, a combination of methods is required to better characterize overall physical literacy progress. Some qualitative measures/assessments captured interactions with the environment and interactions with other individuals, which cannot be captured in quantitative research. In both qualitative and quantitative measures/assessments, children aged under 12 years and PE lessons were the dominant age range and environment to measure/assess physical literacy.

An implication for theory development within physical literacy is the need for researchers to declare their definition and philosophical standpoint whilst undertaking empirical research, to ensure alignment between the definition, philosophy, and measure/assessment adopted. Identifying a philosophical standpoint would enable researchers to “operationalize the related construct of physical literacy and establish meaningful, measureable differences” [4]. Without this, it is problematic for practitioners to fully decipher how best to apply and measure/assess the concept of physical literacy. Hence, consideration of the definition and philosophical groundings is required to ensure the methods of measuring/assessing physical literacy are suitable for research purposes, i.e., to identify the effectiveness of an intervention. Many of the measures/assessments, across all domains, require a level of expertise while administrating and analyzing, which may be problematic in school and community-based settings [12, 86]. Further, some measures/assessments may require accompanying training to ensure the pedagogical processes are appropriate. For example, in an educational context, teachers conducting measures/assessments of the physical domain promoting a comparative environment may be detrimental to a pupil’s physical literacy progress. Practitioners should first concentrate on the process of applying high-quality pedagogy to reflect their definition and philosophy of physical literacy, before assessing the outcomes (measures/assessments) [109]. The pedagogical principles consistent with high-quality practical delivery in PE have been identified by Morgan [110] using Ames’ [100] interpretation of Epstein’s [111] work on developing effective learners. Specifically, Morgan [110] argues that the pedagogical principles identified by creating a mastery motivation are consistent with the holistic concept of physical literacy and high-quality PE.

Similarly, to apply high-quality pedagogy to foster physical literacy, practitioners should also consider creating a caring climate [112], empowering climate [113], and motivational atmosphere [114].

Recommendations for future research include a need for more empirical research on the concept of physical literacy; essentially, there is a need for more research that is open about the definition and philosophical approach used and theories tested. Future research should measure/assess beyond the constructs of physical proficiencies, and aim to measure/assess physical literacy from a more holistic perspective. Further, research across all ages and different environments is required; current research is predominantly constrained to children and youth in PE lesson settings with a minority measuring/assessing physical literacy in young adults, adults, and the elderly.

## Electronic supplementary material

Below is the link to the electronic supplementary material.

Supplementary material 1 (DOCX 649 kb)

Supplementary material 2 (DOCX 28 kb)
