# Supplementary material for: ‘Measuring’ Physical Literacy and Related Constructs: A Systematic Review of Empirical Findings
Source: Sports Med. 2017 Nov 15;48(3):659–82. doi: 10.1007/s40279-017-0817-9 (PMC5808047; doi:10.1007/s40279-017-0817-9)
Supplement: Supplementary file 1 — Supplementary material 1 (DOCX 649 kb) [file 40279_2017_817_MOESM1_ESM.docx]

Electronic Supplementary Material Appendix S1

Full electronic search strategy for SPORTDiscus

1.
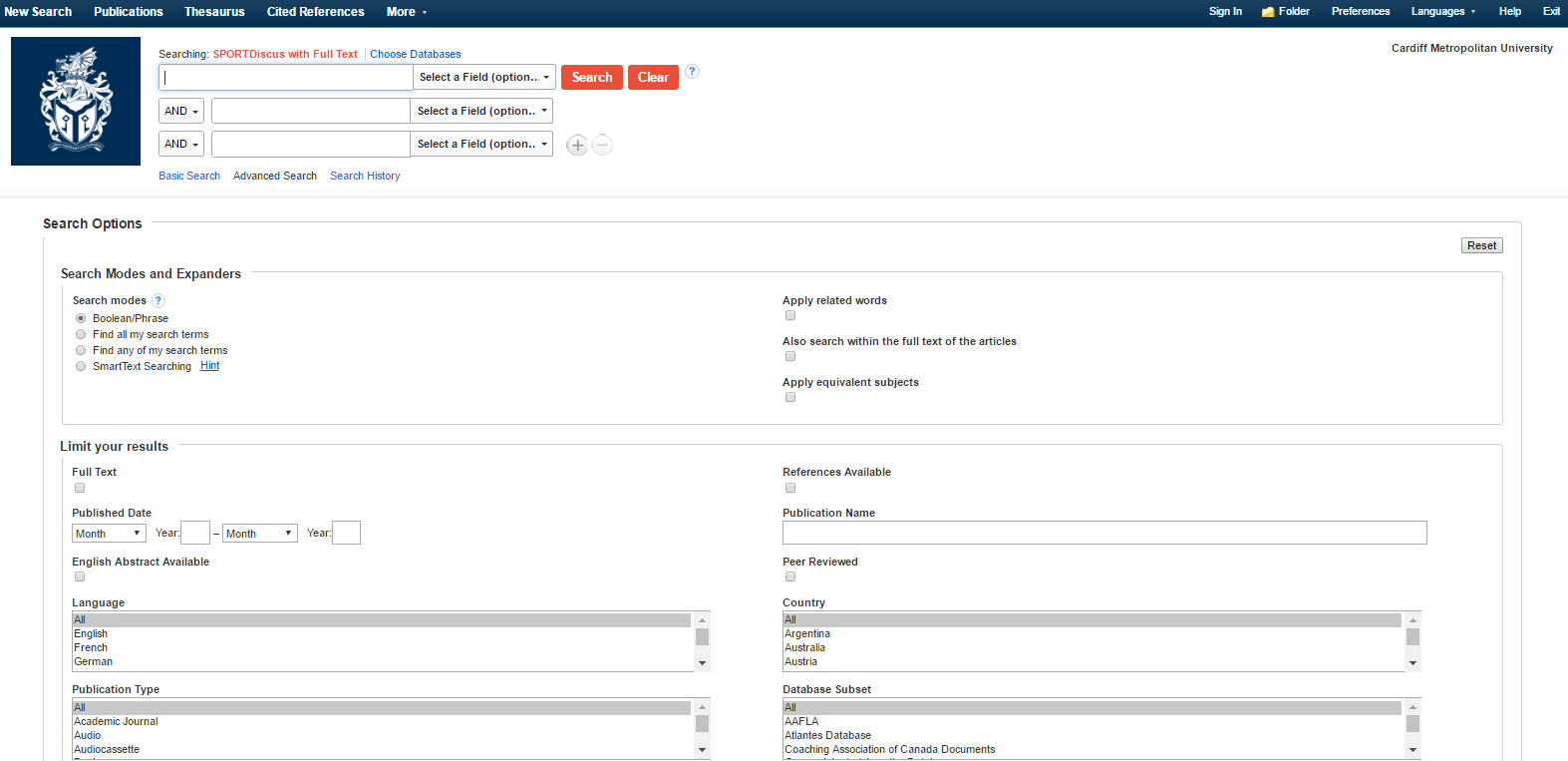
Log into database (SPORTDiscus).
2. Insert keywords into search field (i.e. “physical literacy”). Ensure inverted commas are inserted to the term “physical literacy”.


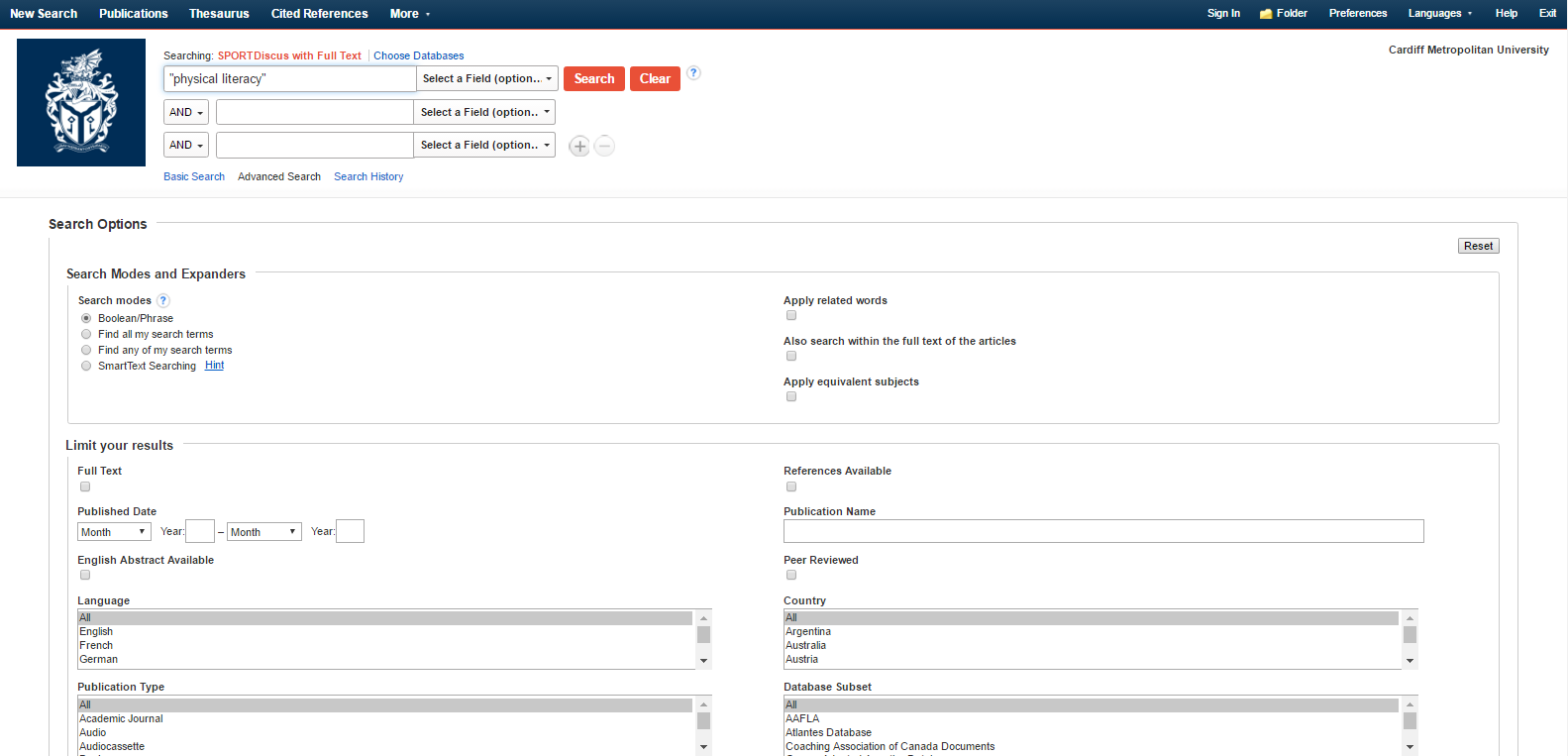


1. Select the ‘Peer reviewed’ and ‘English language’ limits. Press search to view results.


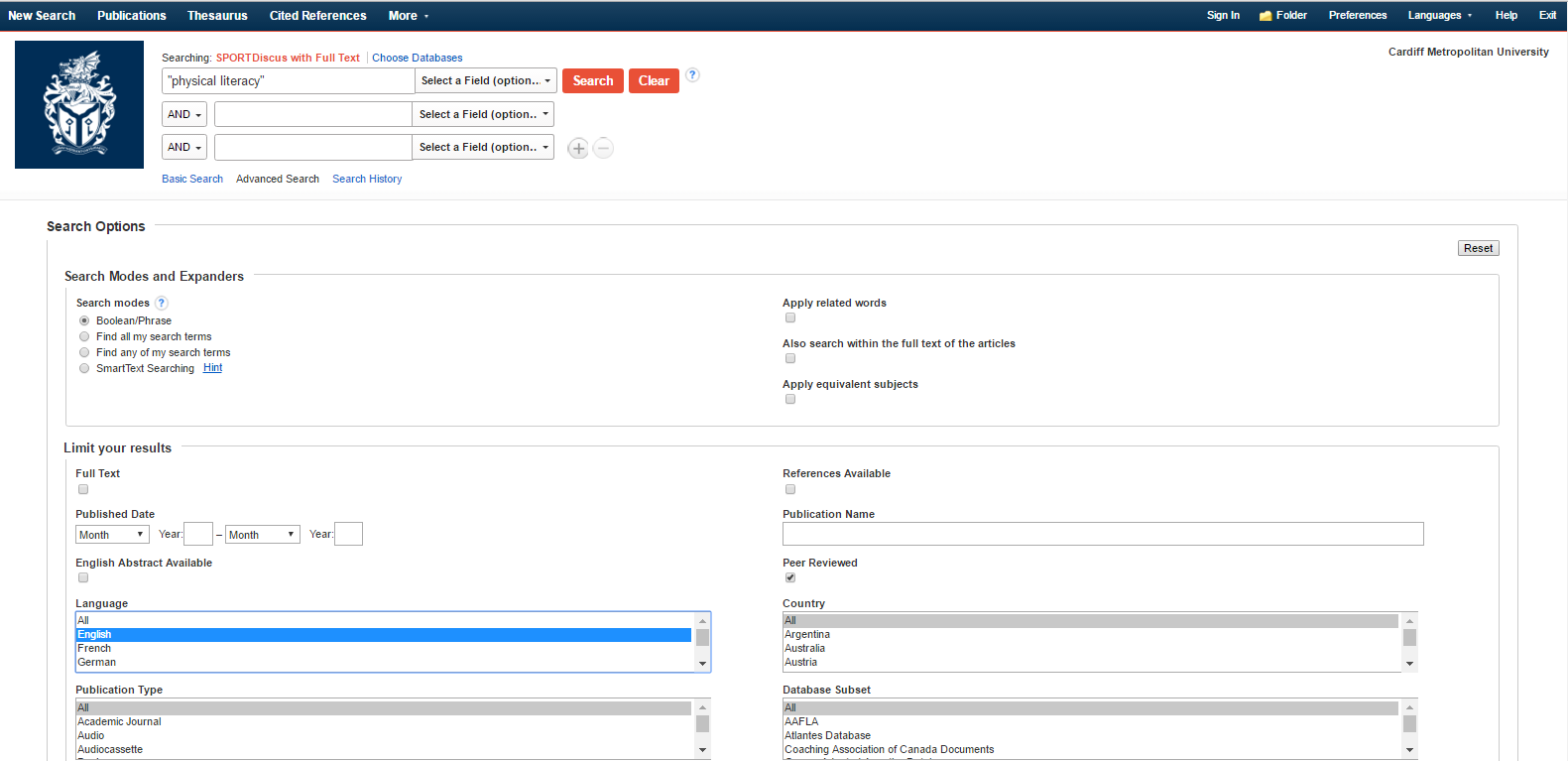


1. The number of results will appear (i.e. 114 results).
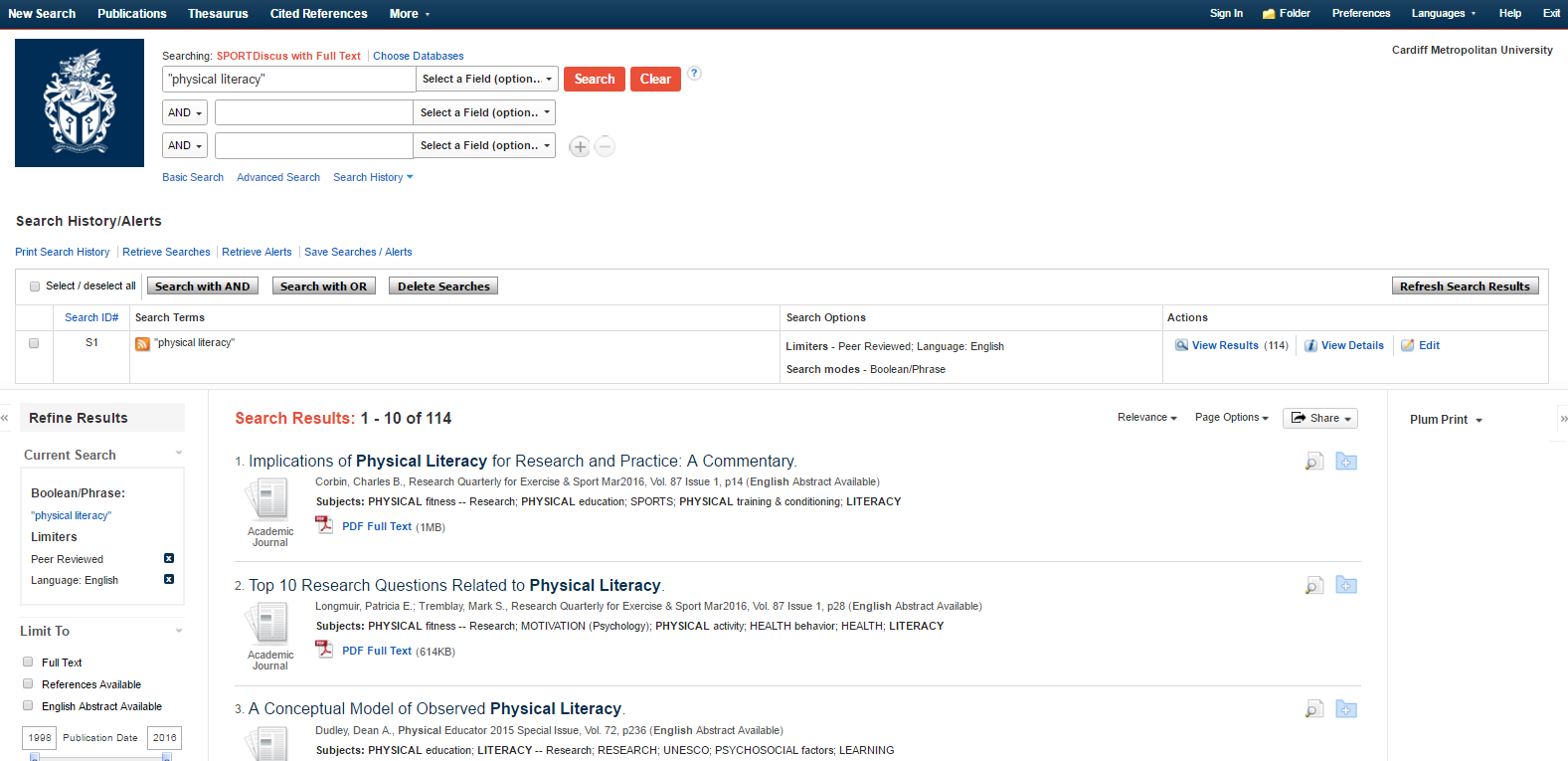

2. Copy and paste each result (reference) into a Microsoft Word document.


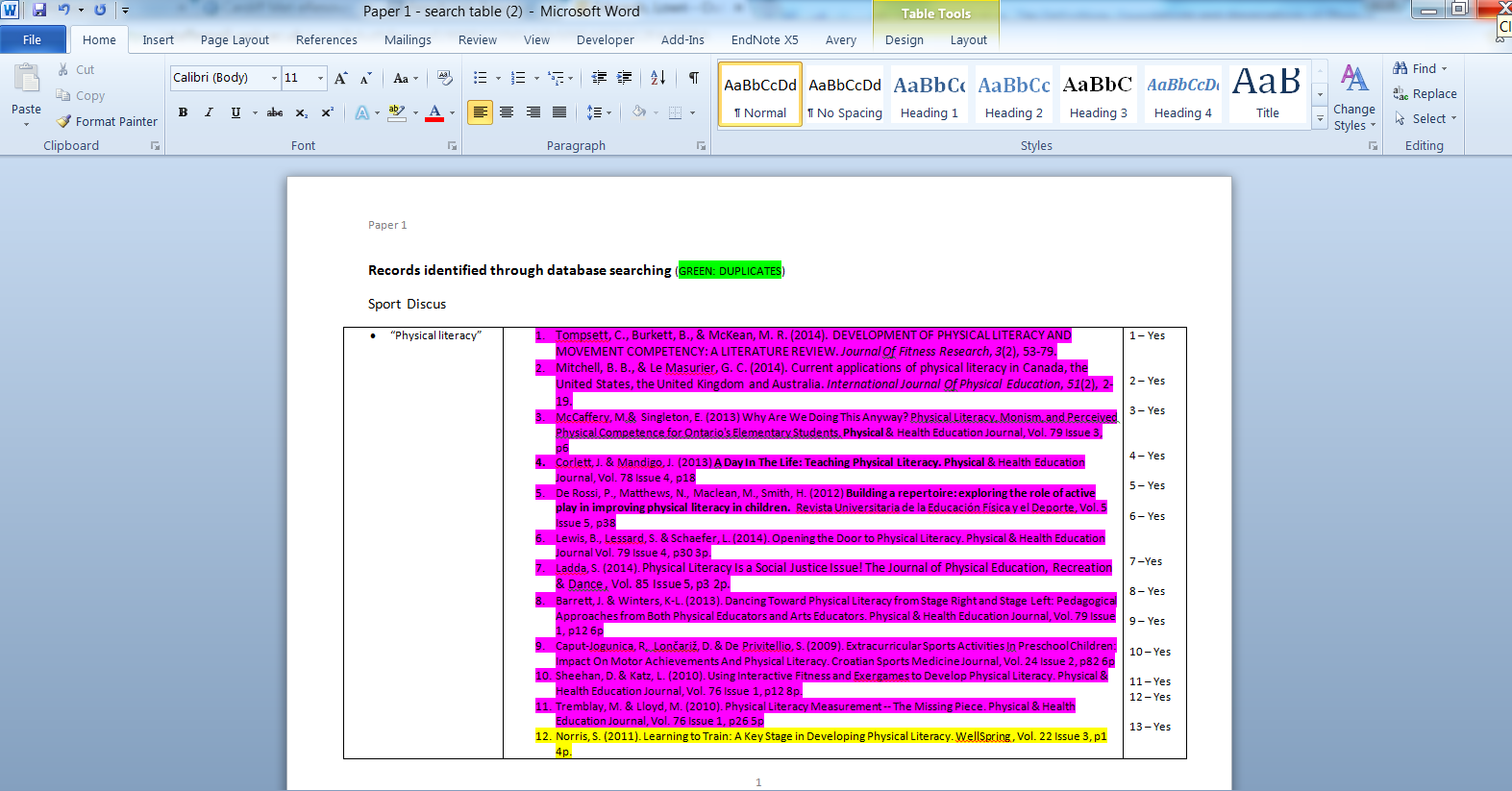


1. Repeat the same procedure with all key search terms (i.e. “physical literacy” AND measurement).
2. Ensure the duplicated papers are highlighted in a different color in the Word document.
3. After duplicate papers are removed, use the eligibility criteria (inclusion and exclusion criteria) to select papers for the analysis.
4. Repeat process consistently with other databases (MEDLINE [via PubMed], Scopus, ScienceDirect, and Education Research Complete).
